# Construction of Graphene Oxide Probes Loaded with Antisense Peptide Nucleic Acid and Doxorubicin for Regulating Telomerase Activity and Inducing Apoptosis of Cancer Cells

**DOI:** 10.3390/bios15060337

**Published:** 2025-05-26

**Authors:** Yanyan Zhu, Qinghong Ji, Min Hong

**Affiliations:** 1 School of Municipal and Environmental Engineering, Shandong Jianzhu University, Jinan 250101, China; zhuyanyan@sdjzu.edu.cn; 2 School of Chemistry and Chemical Engineering, Liaocheng University, Liaocheng 252059, China; 17861826032@163.com

**Keywords:** graphene oxide, peptide nucleic acid, doxorubicin, telomerase, gene regulation, cell apoptosis

## Abstract

In this study, we developed a multifunctional graphene oxide (GO)-based nanoprobe co-loaded with antisense peptide nucleic acid (PNA) and the chemotherapeutic agent doxorubicin (DOX). The nanoplatform was strategically functionalized with folic acid ligands to enable folate receptor-mediated tumor targeting. Upon cellular internalization, the antisense PNA component selectively hybridized with human telomerase reverse transcriptase (hTERT) mRNA through sequence-specific recognition, inducing structural detachment from the GO surface. This displacement restored the fluorescence signal of previously quenched fluorophores conjugated to the PNA strand, thereby enabling the real-time in situ detection and quantitative fluorescence imaging of intracellular hTERT mRNA dynamics. The antisense PNA component effectively reduced the hTERT mRNA level and downregulated telomerase activity via an antisense gene regulation pathway, while the pH-responsive release of DOX induced potent cancer cell apoptosis through chemotherapeutic action. This combinatorial therapeutic strategy demonstrated enhanced anticancer efficacy compared to single-modality treatments, achieving a 60% apoptosis induction in HeLa cells through coordinated gene silencing and chemotherapy. This study establishes GO as a promising dual-drug nanocarrier platform for developing next-generation theranostic systems that integrate molecular diagnostics with multimodal cancer therapy.

## 1. Introduction

Telomerase, a ribonucleoprotein complex classified as a reverse transcriptase, is constituted by both RNA and protein components [1]. Notably, this enzyme demonstrates a distinct expression pattern across cell types—maintaining low or undetectable levels in most normal human somatic cells, while being markedly upregulated in approximately 85% of malignant tumor cells [2]. This differential expression pattern positions telomerase as a promising therapeutic target for anticancer strategies. The human telomerase holoenzyme comprises three core components: telomerase RNA (hTR), telomerase-associated protein 1 (hTP1), and the catalytic subunit, telomerase reverse transcriptase (hTERT) [3]. Extensive studies have established that hTERT serves as the rate-limiting determinant of telomerase activity. Mechanistically, experimental evidence demonstrates that the targeted suppression of hTERT mRNA expression through gene silencing technologies effectively inhibits telomerase activity in malignant cells, ultimately leading to the induction of tumor cell apoptosis [4,5].

Antisense oligonucleotide (ASO) technology represents a prominent gene silencing strategy in molecular therapeutics. Conventional ASOs are single-stranded DNA/RNA constructs typically comprising 13–25 nucleotide units [6,7]. Through rational chemical modifications including locked nucleic acid (LNA) and peptide nucleic acid (PNA) integrations [8,9,10,11,12], researchers can enhance three critical pharmacological properties: (1) nuclease resistance for improved biostability, (2) target mRNA binding affinity through structural optimization, and (3) sequence selectivity, while modulating immunogenic potential. Notably, experimental evidence demonstrates that PNAs can effectively bind telomerase RNA (hTR) in cell lysate systems, achieving significant telomerase inhibition [13]. However, the clinical translation of PNA-based therapeutics faces a fundamental challenge: their inherent inability to passively traverse cellular membranes due to their electroneutral backbone characteristics. This biological barrier necessitates the development of advanced nucleic acid delivery platforms, representing a critical frontier in oligonucleotide therapeutic development.

Doxorubicin (Dox), a cornerstone anthracycline chemotherapeutic agent, remains a first-line broad-spectrum anticancer drug extensively employed in clinical oncology for treating diverse malignancies such as osteosarcoma, hepatocellular carcinoma, breast adenocarcinoma, non-small cell lung cancer, and metastatic prostate cancer [14]. Its antitumor efficacy stems from a dual mechanism of action: (1) intercalation into nuclear DNA duplexes to disrupt the replication/transcription processes and induce apoptosis through DNA damage response pathways [15], and (2) telomerase activity suppression via structural interference with the telomerase RNA component (hTR) [16]. Emerging evidence indicates that the latter mechanism may represent a critical contributing factor to its therapeutic efficacy, particularly in targeting cancer stem cells with elevated telomerase expression.

Graphene oxide (GO), a water-dispersible two-dimensional carbon nanomaterial, exhibits exceptional biomedical compatibility due to its surface-exposed hydrophilic functional groups (carboxyl, hydroxyl, and epoxy) that confer colloidal stability in physiological fluids [17,18]. This unique nano-biointerface characteristic, combined with its extended π-conjugated crystalline lattice and ultrahigh specific surface area (>2600 m^2^/g), enables three synergistic functionalities: (1) a molecular adsorption platform for single-stranded antisense oligonucleotides through π-π stacking interactions, (2) a drug delivery vehicle for chemotherapeutic agents via chemical conjugation or physical adsorption resulting from the hydrogen bond interaction and π-π stacking interaction, and (3) a theranostic hybrid system for tumor marker imaging through surface-enhanced Raman scattering (SERS) effects [19,20]. Dai’s group developed polyethylene glycol (PEG)-functionalized nanoscale GO (NGO-PEG) that achieved high loading efficiency for SN-38 through optimized π-orbital interactions, maintaining drug potency while reducing systemic toxicity [21]. Zhang’s group engineered a pH-responsive GO composite with reversible Schiff base linkages, enabling precise Dox release kinetics (82% payload release at pH 5.0 vs. <10% at pH 7.4) through tumor microenvironment-triggered bond cleavage [22].

GO exhibits a preferential adsorption capacity for single-stranded oligonucleotides while demonstrating a weak affinity for hybridized double-stranded structures. This unique property underpins its principal applications in the fluorescence-based detection of extracellular short-chain DNA oligonucleotides [17], as well as fluorescence detection coupled with in situ imaging of intracellular microRNA [18,23]. Regarding long-chain nucleic acids such as intracellular mRNA, their inherent tendency to form secondary structures within cellular environments facilitates a specific detection mechanism. When hybridization occurs between these structured nucleic acids and fluorescently labeled single-stranded oligonucleotides (serving as response nucleic acid sequences) immobilized on GO composite probes, the resulting duplex formation induces the desorption of the fluorescent probes from the GO surface, thereby restoring the quenched fluorescence signal [24]. This fundamental principle enables both fluorescence detection and spatial mapping of mRNA within biological systems. Furthermore, the strategic design of the response nucleic acid sequence as an antisense counterpart targeting specific mRNA sequences permits its additional functionality in gene silencing applications. A notable implementation of this approach was demonstrated by Kim’s research group, who developed a PEG-functionalized GO-PNA delivery platform capable of achieving targeted mRNA gene silencing in tumor cells [25].

**Figure 1:**
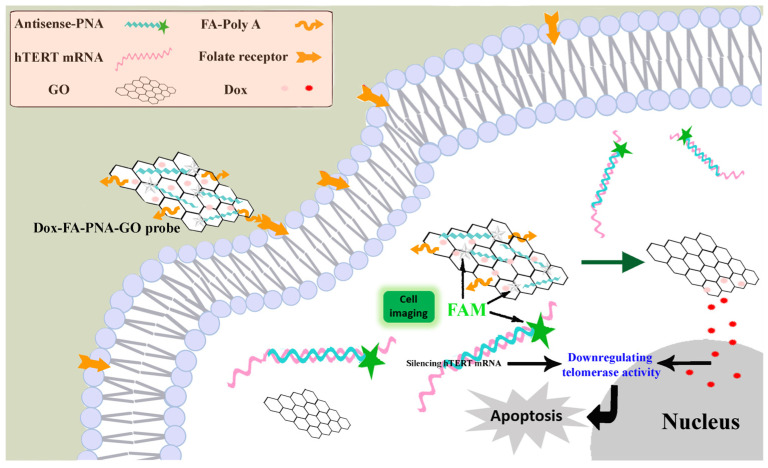
Schematic illustration of the Dox-FA-PNA-GO probe enabling the simultaneous in situ detection, fluorescence imaging, and gene silencing of intracellular hTERT mRNA, integrated with tumor-targeted Dox delivery to achieve downregulation of telomerase activity and further induce apoptosis of cancer cells.

Building upon previous research, we developed a graphene oxide-based probe (Dox-FA-PNA-GO) co-loaded with three functional components: a FAM-labeled antisense peptide nucleic acid (Antisense-PNA), a folic acid (FA)-conjugated oligoadenylate (FA-polyA), and Dox. As illustrated in Figure 1, the intrinsic fluorescence of the FAM fluorophore conjugated to the Antisense-PNA underwent efficient quenching through energy transfer to GO. The design exploited the differential expression of folate receptors, which are minimally expressed on normal cells but overexpressed on cancer cells [26,27], using folic acid as a targeting ligand for tumor-selective recognition. Following cellular internalization, the Antisense-PNA component specifically hybridized with its complementary hTERT mRNA target, forming stable PNA-RNA duplexes. This molecular recognition event triggered the dissociation of Antisense-PNA from the GO substrate, consequently restoring FAM fluorescence emissions—a mechanism enabling the real-time detection of intracellular hTERT mRNA. The Antisense-PNA sequence, designed as a perfect antisense complement to hTERT mRNA, executed dual functions: (1) through classical antisense oligonucleotide action, it induced mRNA silencing via hybridization-blocked translation, effectively downregulating hTERT protein expression to suppress telomerase activity and initiate apoptotic pathways; (2) simultaneously, the acidic tumor microenvironment promoted the controlled release of co-loaded Dox molecules [20,21,22], creating a synergistic therapeutic modality that amplified telomerase inhibition while directly inducing cancer cell death through chemotherapeutic action and allowing for the evaluation of the synergistic apoptosis-inducing potential between gene therapy and chemotherapy modalities.

## 2. Materials and Methods

### 2.1. Apparatus

UV-vis absorption measurements were conducted on a UV-750 spectrophotometer (PerkinElmer, Waltham, MA, USA). Transmission electron microscopy (TEM) imaging was performed with a JEM 2100 system (JEOL Ltd., Tokyo, Japan) at a 200 kV acceleration voltage, using copper grids for sample deposition. Atomic force microscopy (AFM) characterization was performed using a MULTIMODE 8 Solver P47 system (NT-MDT, Moscow, Russia). Particle size distribution and zeta potential measurements were obtained using a Malvern ZETASIZER NANOZSP instrument (Malvern, UK). Fluorescence spectra were recorded using an F-7000 spectrophotometer (Hitachi, Tokyo, Japan). Confocal laser scanning microscopy (CLSM) observations were acquired on a ZEISS LSM 880 microscope. Cellular fluorescence was quantified using a Guava easyCyte 6–2L flow cytometer (Millipore, Burlington, MA, USA). Cellular viability assessments via MTT assay were measured with an ELx808 microplate reader (BioTek, Winooski, VT, USA). Quantitative reverse transcription PCR (qRT-PCR) analysis was implemented using a QuantStudio 5 system (Applied Biosystems, Waltham, MA, USA). 

### 2.2. Reagents

Sodium chloride (NaCl), disodium hydrogen phosphate (Na_2_HPO_4_), potassium chloride (KCl), and potassium dihydrogen phosphate (KH_2_PO_4_) were purchased from Shanghai Chemical Reagent Company. The following reagents were obtained from Sigma-Aldrich (Burlington, MA, USA): 1-(3-dimethylaminopropyl)-3-ethylcarbodiimide hydrochloride (EDC), N-hydroxysuccinimide (NHS), folic acid, 3-[(3-cholamidopropyl) dimethylammonio]-1-propanesulfonate (CHAPS), tris(2-carboxyethyl)phosphine hydrochloride (TCEP), ethylene glycol-bis(2-aminoethyl ether)-N,N,N′,N′-tetraacetic acid (EGTA), phenylmethylsulfonyl fluoride (PMSF), 3-(4,5-dimethylthiazol-2-yl)-2,5-diphenyltetrazolium bromide (MTT), MgCl_2_ solution (1 mol/L), ethylenediaminetetraacetic acid (EDTA), Tris-HCl, and Dox. Gel electrophoresis loading buffer was sourced from Beijing Solarbio Science & Technology Co., Ltd. (Beijing, China). Natural graphite powder (200 mesh, 99.9995%) was acquired from Alfa Aesar China Chemical Co., Ltd. (Shanghai, China). The Human TERT ELISA Kit was procured from Innovation Beyond Limits (Berlin, Germany). All other chemicals were analytical grade. The experimental buffers consisted of phosphate-buffered saline (PBS, pH 7.4: 136.7 mM NaCl, 2.7 mM KCl, 8.72 mM Na_2_HPO_4_, 1.41 mM KH_2_PO_4_).

To investigate the potential dissociation of Antisense-PNA from the GO surface through complementary hybridization, Target-DNA was employed as a fully complementary sequence to facilitate duplex formation. Specifically, 10T-Target-DNA constructs—designed with 10 thymine residues extending both termini of the native Target-DNA—were engineered to mimic the extended single-stranded structure characteristic of hTERT mRNA. Concurrently, NH_2_-Poly A oligonucleotides were utilized for dual functionality: their intrinsic adsorption capability on GO surfaces enabled effective substrate anchoring, while their exposed amine groups served as conjugation sites for folic acid immobilization. PNA was custom-synthesized by Ningbo Kangbei Biochemical Co., Ltd. (Ningbo, China). All deoxyoligonucleotides were commercially synthesized and HPLC-purified by Sangon Biotech (Shanghai, China), with all sequences detailed in Table 1. 

### 2.3. Experimental Sections

#### 2.3.1. Preparation of FA-PNA-GO Probe, PNA-GO Probe, and Dox-FA-PNA-GO Probe

Single-layer GO was initially synthesized using established protocols [28]. Briefly, we mixed graphite (0.3 g) with concentrated sulfuric acid (2.4 mL) in a 100 mL flask at low temperature (0–5 °C), slowly added KMnO_4_ (1.5 g), and stirred to form a pre-oxidation system. Then, we gradually raised the temperature to a medium temperature (about 35 °C) and a high temperature (about 90 °C) for staged temperature-controlled oxidation, promoting the embedding of oxygen-containing functional groups (such as epoxy and carboxyl groups) between graphite layers. After the reaction was complete, deionized water (25 mL) was added to dilute, and H_2_O_2_ was dropped to terminate the reaction. Impurities were removed by centrifugation, acid washing, and water washing, and finally, single-layer GO was obtained by ultrasonic exfoliation.

The FA-PNA-GO probe was then constructed through the following the reference method: First, folic acid was reacted with the amino group on NH_2_-Poly A to obtain the product FA-Poly A. Specifically, 3.2 mg EDC and 4.8 mg NHS were added to 7.91 mL of MES buffer containing 6 μmol/L folic acid, activated by stirring (5 min, room temperature), followed by the addition of 10 μL β-mercaptoethanol to neutralize excess EDC. Then, 80 μL NH_2_-Poly A (300 μmol/L) was introduced and reacted for 4 h (room temperature) to yield FA-Poly A (3 μmol/L). Next, a 200 μL GO suspension (1.124 mg/mL) was dispersed in 2.73 mL Tris-HCl buffer (20 mM, pH 7.4) with 5 min of sonication. Subsequently, 15 μL Antisense-PNA (1 μmol/L) was incubated with GO for 5 min, followed by the addition of 50 μL FA-Poly A (3 μmol/L), and the reaction continued at room temperature for 5 min. Finally, the mixture was centrifuged (15,000 rpm, 20 min) to remove unbound components, and the precipitate was freeze-dried. The final FA-PNA-GO probe (1.12 mg/mL in Tris-HCl) demonstrated a 91% synthesis yield (assuming full component adsorption) and was stored at 4 °C for subsequent experiments.

The preparation of the PNA-GO probe followed a procedure similar to the aforementioned synthesis, with the exception of the fact that exclusively Antisense-PNA was incorporated during the preparation phase. Subsequently, the FA-PNA-GO probe was combined with Dox (2 μmol/L) and allowed to incubate for 24 h. Following centrifugation to remove the supernatant, the resulting precipitate was reconstituted in 200 μL of Tris-HCl buffer solution (20 mM, pH 7.4). The final Dox-FA-PNA-GO probe stock solution was preserved at 4 °C for subsequent experimental applications.

#### 2.3.2. Fluorescence Quenching of GO on Antisense-PNA and Fluorescence Recovery of FA-PNA-GO Probe Triggered by Target-DNA

A 200 μL reaction mixture containing varying concentrations of graphene oxide (GO: 0, 2.5, 5, 7.5, 10, 15, 20, and 30 μg/mL) and Antisense-PNA (200 nmol/L) was prepared and incubated for 16 h. The fluorescence quenching effect of GO on carboxyfluorescein (FAM) modified on Antisense-PNA was subsequently characterized using fluorescence spectroscopy.

Aliquots (0, 1, 2, 3, 4, 5, 10, 20, 30, 40, 60 μL) of 10T-Target-DNA stock solution (1 μmol/L) were combined with the FA-PNA-GO probe (224 μg/mL, 6.7 μL), followed by supplementation with Tris-HCl buffer (20 mM, pH 7.4) to achieve a final reaction volume of 200 μL. The reaction system was incubated at 37°C for 8 h, after which fluorescence intensity measurements were performed. For fluorescence recovery time optimization, five replicate systems containing 200 μL of 10T-Target-DNA (200 nmol/L) mixed with the FA-PNA-GO probe (7.5 μg/mL) were prepared. Fluorescence quantification was conducted at predetermined time intervals to monitor temporal response characteristics.

#### 2.3.3. Determination of Dox Encapsulation Efficiency and Loading Capacity

A standard series of Dox solutions in Tris-HCl buffer (20 mM, pH 7.4) with graded concentrations (0.1, 0.5, 1, 1.5, 2, 2.5, 3 µmol/L) were formulated, and their fluorescence spectra were acquired. A calibration curve was established using the fluorescence intensity at 592 nm. For drug-loading analysis, 3.4 μL of FA-PNA-GO probe suspension (1.12 mg/mL) was blended with 334 μL of Dox solution (3 µmol/L), then diluted with 162.6 μL Tris-HCl buffer to yield a 500 μL system containing 2 µmol/L Dox and 7.5 μg/mL GO. Following a 24 h incubation period, the supernatant was harvested using Amicon ultrafiltration centrifugal devices (10 kDa MWCO) and analyzed by fluorescence spectroscopy. The concentration of free Dox was quantified against the established calibration curve. The drug encapsulation efficiency and loading capacity were calculated through the following equations:

Encapsulation efficiency (%) = [(W_total_ − W_free_)/W_total_] × 100 (1)

Loading capacity (%) = [(W_total_ − W_free_)/W_probe_] × 100 (2)

where W_total_ represents the total mass of Dox added to the probe preparation solution, W_free_ is the mass of unbound Dox measured in the supernatant, and W_probe_ is the mass of the prepared Dox-FA-PNA-GO probe.

#### 2.3.4. Cell Culture

Human cervical carcinoma cells (HeLa), hepatocellular carcinoma cells (HepG2), breast adenocarcinoma cells (MCF-7), osteosarcoma cells (U2OS), and normal mammary epithelial cells (HBL-100) were maintained in Dulbecco’s Modified Eagle Medium (DMEM, Gibco, Billings, MT, USA) supplemented with 10% (*v/v*) fetal bovine serum (FBS), 100 µg/mL penicillin, and 100 µg/mL streptomycin under standardized culture conditions (5% CO_2_, 37 °C). Cellular density was quantified using an automated cell counter. All cell lines were purchased from the Cell Bank of the Chinese Academy of Sciences (Beijing, China).

#### 2.3.5. Confocal Fluorescence Imaging

HeLa, HepG2, MCF-7, HBL-100, and U2OS cells (6 × 10^5^ cells/dish) were plated in confocal imaging chambers and allowed to adhere for 12 h. Subsequently, the FA-PNA-GO probe (7.5 μg/mL) was introduced into the culture medium and gently agitated to ensure homogeneous distribution, followed by undergoing a 6 h incubation period. Live-cell imaging was conducted on a laser scanning confocal microscope (LSM 880, Zeiss) equipped with a 488 nm excitation laser to monitor Antisense-PNA fluorescence recovery dynamics.

Parallel experimental setups were established with HeLa and MCF-7 cell lines (6 × 10^5^ cells/well). Cells were exposed to the Dox-FA-PNA-GO probe (7.5 μg/mL) for 6 h under standard culture conditions. Fluorescence signal acquisition was performed through dedicated spectral channels: FAM emission was monitored at 488 nm excitation and Dox fluorescence was tracked using 514 nm excitation. For nuclear counterstaining, DAPI-containing specimens were concurrently imaged through a 405 nm excitation channel.

#### 2.3.6. Determination of Intracellular hTERT Protein Expression Levels

Cell suspensions (0.4 mL, 1 × 10^6^ cells/mL) were plated in 6-well culture plates and maintained under standard conditions (37 °C, 5% CO_2_) for 24 h. After treatment with various GO-based probes (7.5 μg/mL) at 37°C for specified durations, cells were harvested by trypsinization. Aliquots containing 4 × 10^6^ cells were transferred to 1.5 mL microcentrifuge tubes, subjected to two cycles of washing with pre-chilled PBS (0.1 mol/L, pH 7.4), and ultimately resuspended in 200 μL ice-cold CHAPS lysis buffer (10 mmol/L Tris-HCl, pH 7.5; 1 mmol/L MgCl_2_; 1 mmol/L EGTA; 0.1 mmol/L PMSF; 0.5% CHAPS; 10% glycerol). Following 30 min on-ice incubation, lysates were centrifuged at 16,000× *g* (4 °C, 20 min). Clarified supernatants were either immediately diluted to 200 μL for analysis or cryopreserved at −150 °C. Quantitative determination of hTERT protein expression levels was performed using a commercial ELISA kit according to manufacturer protocols.

#### 2.3.7. RNA Extraction and Quantitation of hTERT mRNA by PCR

HeLa cells were exposed to various GO probes (7.5 μg/mL) for specified durations, with untreated controls maintained in parallel. Total RNA isolation was performed using TRIzol™ reagent (TIANGEN Biotech, Beijing, China). First-strand cDNA synthesis was executed with the QuantiNova Reverse Transcription Kit (Qiagen, Hilden, Germany) under programmed thermal cycling: 45 °C for 2 min (genomic DNA elimination), 25 °C for 3 min (primer annealing), 45 °C for 10 min (reverse transcription), and 85 °C for 5 min (enzyme inactivation), followed by a 4 °C hold. Quantitative real-time PCR analysis was conducted on a QuantStudio™ 5 System (Applied Biosystems, Waltham, MA, USA) with the following amplification protocol: initial denaturation at 95 °C for 2 min, 40 cycles of 95 °C for 15 s (denaturation), 55 °C for 15 s (annealing), and 72 °C for 1 min (extension). Primer sequences were as follows: hTERT: Forward 5′-CGGAAGAGTGTCTGGAGCAA-3′, Reverse 5′-CACGACGTAGTCCATGTTCA-3′ and GAPDH (housekeeping gene): Forward 5′-CTCAGACACCATGGGGAAGGTGA-3′, Reverse 5′-ATGATCTTGAGGCTGTTGTC-ATA-3′.

#### 2.3.8. Cellular Viability Study

HeLa cells were plated in 96-well microplates at a density of 1 × 10^5^ cells/well and allowed to adhere for 24 h under standard culture conditions. Following adherence, cells were challenged with eight experimental formulations: (1) pristine GO (7.5 μg/mL), (2) free FA-Poly A (3 μmol/L), (3) Antisense-PNA (3 μmol/L), (4) PNA-GO probe (7.5 μg/mL), (5) FA-PNA-GO probe (7.5 μg/mL), (6) FA-Control-PNA-GO probe (7.5 μg/mL), (7) Dox-FA-PNA-GO probe (7.5 μg/mL) and (8) Dox (0.8 μg/mL), each incubated for designated time intervals. Post-treatment cellular viability was quantified via an MTT colorimetric assay [29], with viability expressed as a percentage relative to untreated controls.

#### 2.3.9. Flow Cytometric Analysis of Cell Apoptosis Induced by FA-PNA-GO Probes

HeLa cells were subjected to time-course experiments with either PBS (control) or FA-PNA-GO probes (7.5 μg/mL). Following treatment, culture medium was aspirated and cells were trypsinized, pelleted by centrifugation (300× *g*, 5 min), and washed twice with ice-cold PBS (pH 7.4). Apoptotic cell populations were quantified using an Annexin V-FITC Apoptosis Detection Kit (Beyotime) according to manufacturer specifications. Flow cytometric analysis was performed on a Guava easyCyte 6-2L flow cytometer (Millipore, Burlington, MA, USA) with standardized acquisition parameters (5000 events per sample).

## 3. Results and Discussion

### 3.1. Characterization of GO and FA-PNA-GO Probe

**Figure 2:**
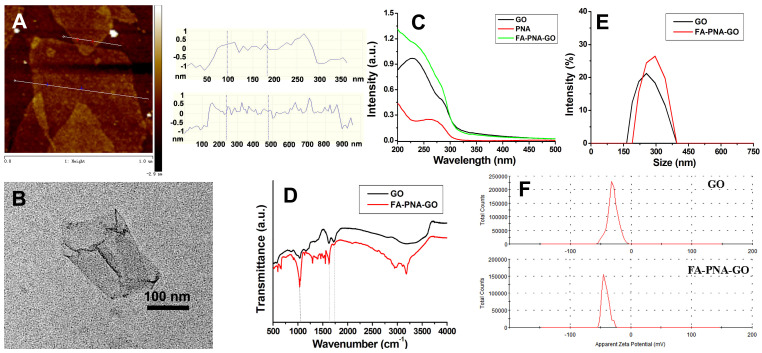
AFM images of GO with corresponding line scan profile (**A**); TEM image of GO (**B**); UV−vis spectra of GO, PNA, and the FA-PNA-GO probes (**C**); and IR spectra (**D**), size distribution (**E**)**,** and zeta potential (**F**) of GO and the FA-PNA-GO probes.

The morphological features of graphene oxide (GO) were analyzed through AFM (Figure 2A) and TEM (Figure 2B), collectively validating the synthesis of monolayer GO sheets. UV-Vis spectral analysis identified two distinct absorption bands for GO (Figure 2C): a prominent peak at 230 nm assigned to π-π* electronic transitions in aromatic C=C bonds, and a weaker shoulder near 295 nm associated with the n→π* transitions of carbonyl (C=O) groups. Notably, the FA-PNA-GO probe displayed a composite absorption profile integrating the 260 nm signature of Antisense-PNA with characteristic GO absorption features, confirming successful surface modification. Infrared (IR) spectroscopy (Figure 2D) further corroborated the structural integrity of GO and its functionalized derivatives. A strong and broad absorption peak near 3400 cm^−1^ corresponded to the stretching vibration of -OH, while the peak around 1750 cm^−1^ was attributed to the stretching vibration of the C=O bond in the carboxyl groups of GO. The peak at 1630 cm^−1^ corresponded to the bending vibration of C-OH, and the peak around 1100 cm^−1^ was due to the deformation vibration of C-O-C in GO. Additional absorption bands in the FA-PNA-GO system originated from surface-anchored deoxyoligonucleotides and Antisense-PNA moieties. Particle size distribution and surface charge analysis via dynamic light scattering (DLS, Figure 2E,F) revealed comparative insights: unmodified GO exhibited an average hydrodynamic diameter of ~240 nm with a zeta potential of −30 mV, whereas the FA-PNA-GO probe showed a marked increase in particle size (~300 nm) accompanied by a pronounced negative potential shift (−45 mV). These alterations stemmed from the electrostatic binding of negatively charged nucleic acid components to the GO substrate.

### 3.2. Fluorescence Spectroscopic Analysis of GO-Mediated Antisense-PNA Adsorption and Fluorophore Quenching

**Figure 3:**
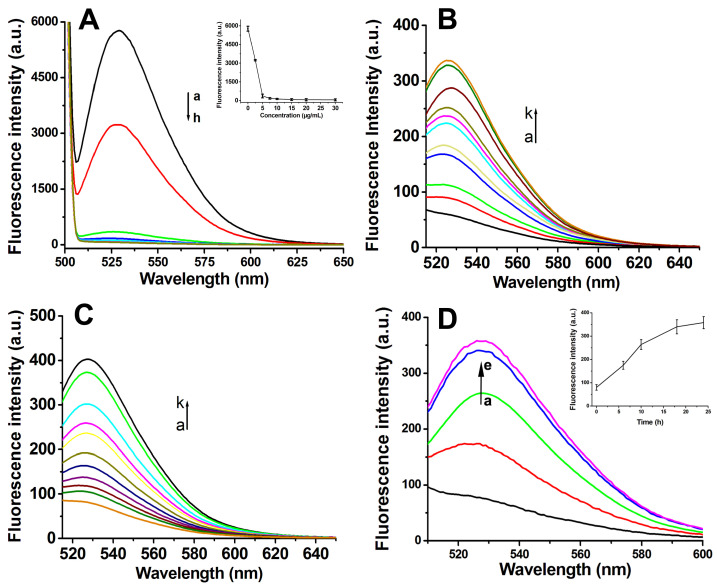
(**A**) Fluorescence spectra of the mixtures of GO at different concentrations (a–h: 0, 2.5, 5, 7.5, 10, 15, 20, and 30 g/mL) and Antisense-PNA (200 nmol/L) after incubation for 16 h. Fluorescence spectra of PNA-GO probes treated with different concentrations of Target-DNA (**B**) or 10T-Target-DNA (**C**) (a–k: 0, 5, 10, 15, 20, 25, 50, 100, 150, 200, and 300 nmol/L) for 18 h. (**D**) Fluorescence spectra of the PNA-GO probe after incubation with 10T-Target-DNA for different times (a–e: 0, 6, 10, 18, and 24 h). Insert: Plot of fluorescence intensity vs. incubation time.

Fluorescence spectroscopy was employed to systematically investigate the adsorption capacity of GO for Antisense-PNA and its concomitant fluorescence quenching effect on the conjugated FAM fluorophore. Quantitative analysis revealed a concentration-dependent fluorescence attenuation pattern, where the incremental addition of GO (0–7.5 μg/mL) induced the progressive diminution of the FAM emission intensity. Beyond this critical threshold (7.5 μg/mL), the fluorescence intensity plateaued at minimum detectable levels (Figure 3A). This optimized concentration was strategically selected to achieve dual objectives: (1) ensuring >99% fluorescence quenching efficiency in the functionalized composite system, and (2) eliminating potential interference from unbound GO excess in downstream biological applications.

### 3.3. Fluorescence Spectroscopic Analysis of PNA-GO and FA-PNA-GO Probes in Response to Complementary Targets

**Figure 4:**
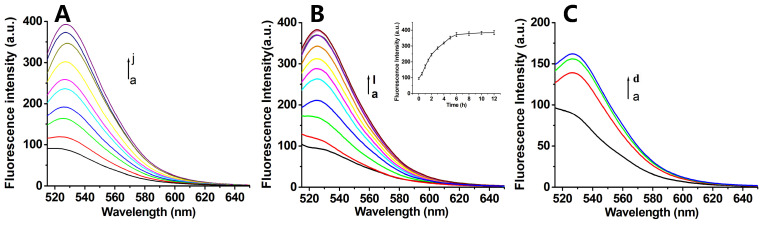
(**A**) The fluorescence spectra of the FA-PNA-GO probe with different concentrations of 10T-Target-DNA (a–j: 0, 10, 20, 30, 40, 50, 100, 150, 200, 300 nmol/L) for 6 h. (**B**) The fluorescence spectra of the FA-PNA-GO probe with 10T-Target-DNA at different times (a–l: 0, 0.5, 1.0, 1.5, 2.0, 3.0, 4.0, 5.0, 6.0, 8.0, 10.0, 12.0 h), respectively. Insert: Plot of fluorescence intensity vs. incubation time. (**C**) Fluorescence spectra of the FA-PNA-GO probe treated with cell lysates of different numbers of HeLa cells (a–d: 0, 2.4 × 10^4^, 4.8 × 10^4^, 9.6 × 10^4^) for 6 h.

The detection mechanism relied on fluorescence restoration through the target-induced detachment of FAM-labeled Antisense-PNA from the GO surface. To emulate intracellular conditions, we engineered two distinct DNA targets: (1) Target-DNA (full-length complement to Antisense-PNA) and (2) 10T-Target-DNA (extended with 10 thymine residues at both termini). Fluorescence recovery assays with PNA-GO and FA-PNA-GO probes demonstrated concentration-dependent signal restoration for both targets (Figure 3B,C and Figure 4A). Kinetic profiling revealed critical operational differences: The PNA-GO system required prolonged incubation (>18 h, Figure 3D) for detectable signal recovery, rendering it suboptimal for real-time mRNA tracking. Conversely, the FA-PNA-GO probe achieved maximal fluorescence stabilization within 6 h (Figure 4B), indicating enhanced displacement kinetics from FA-Poly A co-modification. We attribute this acceleration to electrostatic shielding effects, where negatively charged FA-Poly A attenuates π-π stacking interactions between GO and Antisense-PNA, thereby facilitating faster probe release upon target hybridization.

To validate the sequence-specific responsiveness of Antisense-PNA-functionalized probes, we engineered a length-matched Control-PNA with a randomized nucleotide composition for comparative testing. Crucially, both PNA-GO and FA-PNA-GO systems incorporating Control-PNA exhibited negligible hybridization responsiveness toward either Target-DNA or 10T-Target-DNA sequences, thereby confirming the critical role of sequence complementarity in probe activation.

To further verify the diagnostic efficacy of the hTERT mRNA detection platform, we performed comparative fluorescence analysis of cell lysates incubated with either the FA-PNA-GO probe or its non-targeting counterpart (FA-Control-PNA-GO). As evidenced in Figure 4C, the experimental group exhibited a cell number-dependent fluorescence enhancement, whereas the control system maintained baseline signal levels. This dose-responsive behavior conclusively demonstrated the probe’s capability to specifically recognize endogenous hTERT mRNA within complex biological matrices.

### 3.4. In Situ Detection, Fluorescence Imaging, and Time Response of Intracellular hTERT mRNA Using the FA-PNA-GO Probes

**Figure 5:**
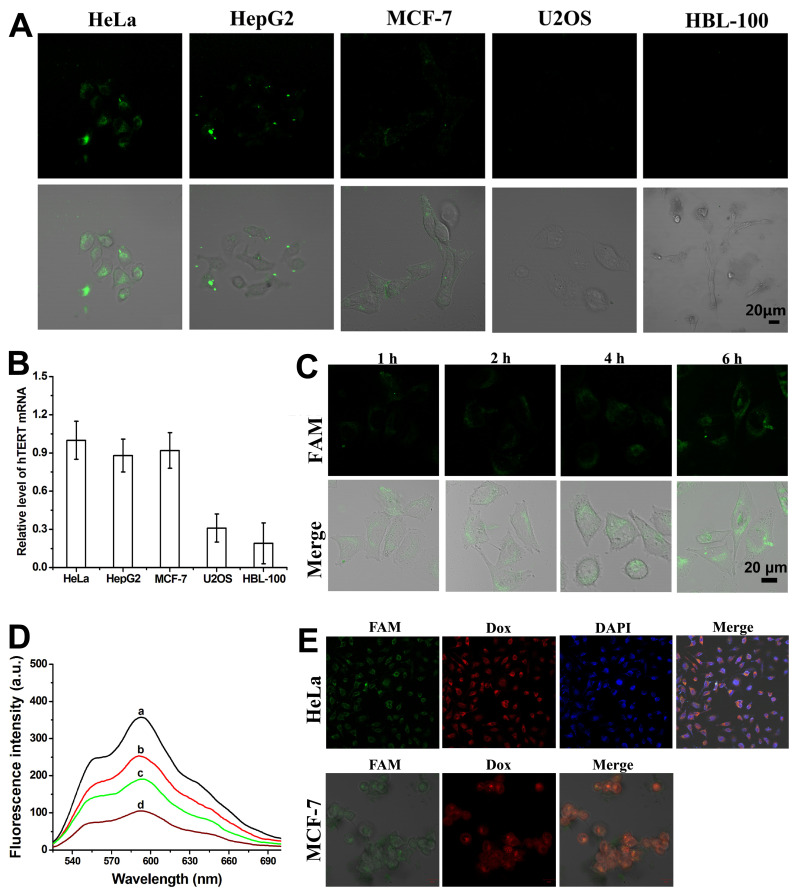
(**A**) Confocal images of HeLa, HepG2, MCF-7, U2OS, and HBL-100 cells after incubation with FA-PNA-GO probes for 6 h: fluorescence field image (above) and merged image of fluorescence field and bright field (below). (**B**) hTERT mRNA level analysis through the qRT-PCR for different cells, using the 2^-ΔΔCT^ method to calculate relative mRNA levels and including GAPDH as an internal control. (**C**) Time-course of CLSM images of HeLa cells incubated with FA-PNA-GO probe. (**D**) Fluorescence spectra of Dox without or with the FA-PNA-GO probe. Fluorescence spectra of Dox alone (2 µmol/L) (a), and the mixture of Dox (2 µmol/L) and the FA-PNA-GO probe (7.5 μg/mL) after reacting for 1 h (b), 6 h (c), and 24 h (d). (**E**) CLSM images of HeLa and MCF-7 cells after incubation with Dox-FA-PNA-GO probes for 6 h.

Studies have demonstrated that telomerase and its corresponding hTERT mRNA exhibit significantly higher expression levels in most cancer cells compared to their low expression in normal cells [30,31]. In this study, we employed the FA-PNA-GO probe for the in situ detection of intracellular hTERT mRNA, selecting four cancer cell lines for experimental validation. The results revealed elevated hTERT mRNA expression in cervical (HeLa), hepatocellular carcinoma (HepG2), and breast cancer (MCF-7) cells, whereas osteosarcoma cells (U2OS) showed minimal expression [32]. As a normal cell control, HBL-100 breast epithelial cells similarly displayed low hTERT mRNA expression. Consistent with previous reports [18,19,20,21,22,29], the FA-PNA-GO probe demonstrated efficient cellular internalization via endocytosis. Subsequent hybridization between the surface-conjugated nucleic acid sequences and intracellular hTERT mRNA triggered the release of FAM-labeled Antisense-PNA from the GO surface, resulting in fluorescence signal recovery. Confocal microscopy analysis (Figure 5A) showed distinct fluorescence signals in HeLa, HepG2, and MCF-7 cells following 6 h of probe incubation, in contrast to the negligible fluorescence observed in U2OS and HBL-100 cells. These findings were corroborated by quantitative PCR measurements of hTERT mRNA expression levels across different cell types (Figure 5B). These collective results confirmed two key findings: (1) the FA-PNA-GO probe specifically recognized intracellular hTERT mRNA, and (2) Antisense-PNA release from the nanoprobe strictly depended on complementary hybridization with target mRNA. Notably, the acidic intracellular environment of U2OS cells failed to induce the nonspecific detachment of Antisense-PNA from the GO surface.

To dynamically monitor intracellular hTERT mRNA expression, we performed time-course confocal imaging following FA-PNA-GO probe administration, thus establishing the temporal response profile of this nanoprobe. Confocal imaging time-course analysis (Figure 5C) revealed detectable fluorescence signals as early as 1 h post-probe administration. The fluorescence intensity showed progressive enhancement with an extended incubation duration, demonstrating a comparable time-dependent activation pattern to that observed in the FA-PNA-GO probe/10T-Target-DNA interaction experiments (Figure 4B).

### 3.5. Design and Characterization of the Dox-FA-PNA-GO Probe

Beyond targeting intracellular hTERT mRNA, we sought to explore the therapeutic potential of gene–drug synergistic therapy for enhanced anticancer efficacy. Using a combinatorial approach, Dox was employed as a model chemotherapeutic agent for proof-of-concept validation. Fluorometric analysis (Figure 5D) demonstrated time-dependent fluorescence attenuation of Dox when co-incubated with the FA-PNA-GO probe, attributed to Förster resonance energy transfer (FRET) between the GO-adsorbed Dox and the nanosystem, resulting in effective fluorescence quenching. This observation confirmed the preserved Dox-loading capacity of the FA-PNA-GO dual-loading system (Antisense-PNA/FA-DNA), with quantitative characterization revealing an 82% encapsulation efficiency and 10.5% drug payload.

### 3.6. Real-Time Tracking of Therapeutic Cargo Release from Dox-FA-PNA-GO Nanoplatform in Cancer Cells

To validate the dual payload release kinetics of Antisense-PNA and Dox from the Dox-FA-PNA-GO complex, live-cell confocal imaging was performed in HeLa and MCF-7 models. Time-lapse imaging (Figure 5E) revealed co-localized fluorescence signals (FAM: 488 nm; Dox: 561 nm) in HeLa cells after 6 h incubation, confirming the synchronous cytoplasmic liberation of both therapeutic components. Nuclear counterstaining with DAPI demonstrated Dox nuclear translocation in subpopulations, indicating functional drug delivery. Here, the release mechanisms diverged between components. Antisense-PNA dissociation originated from target mRNA hybridization-triggered displacement from GO. Dox liberation resulted from pH-responsive desorption in the acidic tumor microenvironment [20,21,22,33,34]. Consistent release patterns were also observed in MCF-7 cells, validating the nanoplatform’s broad applicability across cancer cell types.

### 3.7. Investigating the FA-PNA-GO Probe’s Dual Functionality: hTERT mRNA/Protein Downregulation and Proapoptotic Activity in Cancer Cells

**Figure 6:**
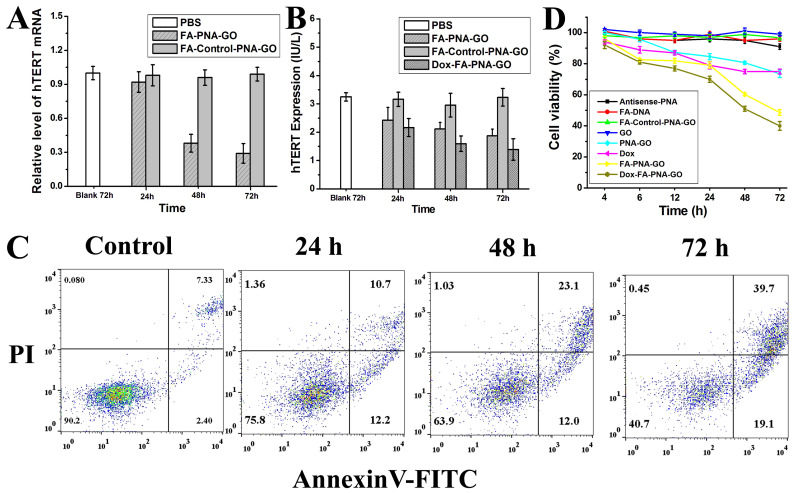
(**A**) hTERT mRNA expression analysis through the qRT-PCR in HeLa cells after treatment with different GO probes for different times. (**B**) The expression of hTERT in HeLa cells treated with GO probes for different times. (**C**) Annexinv-FITC and propidine iodide (PI) staining were used to identify apoptotic and necrotic cells: “Control” represents the flow cytoflex analysis of HeLa cells after treatment with PBS for 72 h; the other images show the data of HeLa cells after being treated by FA-PNA-GO probes for different times. (**D**) Viability of HeLa cells after being treated by different formulas for different times.

Given the established correlation between hTERT mRNA expression and telomerase activation during cellular immortalization, we employed antisense oligonucleotide technology to target intracellular hTERT mRNA, aiming to progressively reduce both hTERT levels and telomerase activity. To evaluate the regulatory capacity of the FA-PNA-GO probe, time-dependent cellular treatments were conducted, followed by the systematic assessment of hTERT mRNA and protein suppression. RT-PCR analysis (Figure 6A) demonstrated the progressive downregulation of hTERT mRNA in probe-treated HeLa cells, showing a statistically significant reduction compared to untreated controls that intensified with prolonged exposure. Complementary quantitative analysis using a commercial hTERT ELISA kit revealed the parallel attenuation of telomerase reverse transcriptase activity (Figure 6B), exhibiting temporal dynamics congruent with mRNA suppression patterns. Notably, control experiments with the FA-Control-PNA-GO probe showed negligible effects on both hTERT mRNA and protein expression profiles.

Building on the confirmed hTERT suppression mechanism mediated by the FA-PNA-GO probe through mRNA downregulation, we next explored the functional correlation between hTERT silencing and probe-induced apoptosis in HeLa cells. Apoptotic dynamics were quantitatively assessed via flow cytometry with Annexin V-FITC/PI dual staining. Vehicle-treated controls (PBS buffer) exhibited baseline apoptosis rates of 7.33% (early) and 2.4% (late), respectively (Figure 6C). Compared to the results after 24 h of treatment with the FA-PNA-GO probe, the proportions of early and late apoptotic cells increased to 23.1% and 12%, respectively, after 48 h of incubation. When the treatment time was further extended to 72 h, the proportions reached 39.7% and 19.1%, respectively. This progressive apoptotic induction confirmed the FA-PNA-GO probe’s potent proapoptotic capacity. Comparative analysis with our previously developed LNA-gold nanoparticle system [35] revealed the enhanced apoptotic efficacy of the FA-PNA-GO probe, attributable to its superior target specificity, folate receptor-mediated precision delivery, and optimized pharmacodynamic profile.

### 3.8. Synergistic Telomerase Suppression by Dox-FA-PNA-GO Nanoplatform 

Next, we further characterized the combinatorial therapeutic effects through telomerase activity profiling. Comparative analysis (Figure 6B) revealed enhanced telomerase inhibition in Dox-FA-PNA-GO-treated HeLa cells relative to the Dox-free FA-PNA-GO system across all timepoints. This amplified suppression demonstrated the cooperative interaction between the antisense oligonucleotide and Dox, synergistically targeting telomerase machinery to potentiate cancer cell apoptosis.

### 3.9. Assessing the Cytotoxic Efficacy of Different Nanosystems: Time-Dependent Modulation of Cellular Viability

To establish the functional linkage between hTERT mRNA level and cancer cell viability, we conducted systematic MTT assays evaluating HeLa cells treated with six experimental conditions: GO, FA-DNA, Antisense-PNA, PNA-GO, FA-PNA-GO, and FA-Control-PNA-GO probes across multiple timepoints. Quantitative analysis revealed negligible cytotoxicity for the GO, FA-DNA, Antisense-PNA, and FA-Control-PNA-GO groups, whereas the PNA-GO and FA-PNA-GO treatments exhibited distinct time-dependent viability reduction (Figure 6D). The FA-PNA-GO system demonstrated dual functional enhancement—folate-mediated targeting improved cellular uptake efficiency, and consequent Antisense-PNA delivery amplified proapoptotic efficacy. Notably, FA-PNA-GO-treated cells showed 48% viability at 72 h, significantly lower than the 73% viability observed with PNA-GO treatment at equivalent concentrations. These findings conclusively establish hTERT mRNA suppression as an effective strategy for inhibiting cancer cell proliferation.

Further, comparative analysis of apoptotic induction capacity (Figure 6D) revealed the superior therapeutic performance of the Dox-FA-PNA-GO probe compared to free Dox and Dox-free FA-PNA-GO systems, with the efficacy enhancement intensifying over time. Quantitative viability assessment demonstrated a marked reduction to 53% in Dox-FA-PNA-GO-treated cells at 48 h, significantly outperforming control groups (free Dox: 78%; FA-PNA-GO: 63%). This time-dependent therapeutic amplification was attributable to progressive Dox release from the nanoplatform, synergizing with Antisense-PNA-mediated gene regulation to enhance cancer cell apoptosis. An extended 72 h treatment further reduced viability to 40%, confirming the sustained combinatorial effect.

## 4. Conclusions

We engineered a multifunctional GO-based nanoplatform co-loaded with three therapeutic components: folate-targeting ligands, Antisense-PNA, and chemotherapeutic Dox. This system exhibited dual-functional capability—simultaneous detection (through in situ hTERT mRNA fluorescence imaging) and dynamic regulation (via mRNA level suppression and telomerase inhibition). Remarkably, the incorporated Dox synergistically amplified telomerase suppression while potentiating apoptotic induction through multimodal therapeutic interplay. This proof-of-concept study validates GO’s unique advantages as a biocompatible nanovector, demonstrating triple payload co-delivery capacity for (a) molecular imaging, (b) antisense gene therapy, and (c) chemotherapeutic intervention, thereby establishing a versatile platform for cancer theranostics.

## Figures and Tables

**Table 1:** The sequences of PNA and different DNA used in the experiments.

Names	Sequences (5′→3′)
Antisense-PNA	FAM-OO-CCAGCCGCCAGCCCT
Control-PNA	FAM-OO-GATGCCGTAAGATCT
Target-DNA	AGGGCTGGCGGCTGG
10T-Target-DNA	TTTTTTTTTTAGGGCTGGCGGCTGGTTTTTTTTTT
NH_2_-Poly A	NH_2_-AAAAAA

## Data Availability

The original contributions presented in this study are included in the article. Further inquiries can be directed to the corresponding authors.
